# A two-microRNA signature as a potential biomarker for early gastric cancer

**DOI:** 10.3892/ol.2014.1797

**Published:** 2014-01-15

**Authors:** GUORONG ZHENG, YIMIN XIONG, WEITIAN XU, YAN WANG, FANG CHEN, ZHIGANG WANG, ZHI YAN

**Affiliations:** 1 Department of Digestive Diseases, Wuhan General Hospital of Guangzhou Command, Wuchang, Wuhan 430070, P.R. China; 2 Department of Medical Laboratory, Wuhan General Hospital of Guangzhou Command, Wuchang, Wuhan 430070, P.R. China; 3 Department of Oncology, Wuhan General Hospital of Guangzhou Command, Wuchang, Wuhan 430070, P.R. China

**Keywords:** gastric cancer, early diagnosis, microRNAs, biomarkers

## Abstract

Gastric cancer (GC) is one of the most common malignant tumors worldwide. No fundamental improvements in the five-year survival rates of patients with GC have been reported due to a low early diagnosis rate. Therefore, the identification of novel biomarkers is urgently required for an early diagnosis of GC. A total of 86 patients were selected for the present study, including 44 patients with early stage GC (T1–T2 according to TNM staging criteria) and 42 normal gastric mucosa samples from non-cancer patients as controls. A total of 18 samples were used for the microRNA (miRNA) microarray experiments, including nine early GC and nine normal gastric mucosa samples. Bioinformatics algorithms, significant analysis of microarray (SAM), top scoring pair (TSP) and statistical receiver operating characteristic curves were used to identify the best signatures. Finally, quantitative PCR was used to validate the candidate biomarkers for early gastric cancer in the test samples (35 cancer and 33 normal samples). Using the SAM algorithm, 14 differential miRNAs were selected as candidate biomarkers. Using the TSP algorithm, hsa-miR-196a and hsa-miR-148a were obtained as a signature to differentiate between the early GC and normal samples. A coincidental result was observed in the test samples. hsa-miR-196a was upregulated and hsa-miR-148a was downregulated in the early GC samples. hsa-miR-196a and hsa-miR-148a have the potential to serve as candidate biomarkers for early GC.

## Introduction

Gastric cancer (GC) is one of the most frequent malignant tumors with a high mortality rate. Almost two-thirds of GC cases occur in developing countries and the incidence in China accounts for ~42% of all the cases (1). Early GC is defined as a gastric carcinoma that is confined to the mucosa and submucosa, irrespective of lymph node involvement and tumor size (2). Early GC has a good prognosis following curative resection; the five-year survival rate is >90% in certain parts of Asia (3,4) and marginally lower in Europe and the United States (5,6). Currently, surgery remains the main option for treating GC. However, the majority of the patients that present with clinical symptoms of GC are diagnosed with advanced GC. The digestive endoscopic technique has significantly improved the early diagnosis rate of GC. In addition, clinical cancer biomarkers, including CEA and CA199, are effective objective indicators for GC diagnosis. However, a misdiagnosis of patients that are negative for the cancer biomarkers and endoscopic diagnosis may occur. Therefore, the identification of novel biomarkers is urgently required for the early diagnosis of GC.

To date, the study of cancer genomics has extensively penetrated into biomedical research and clinical applications. Numerous studies have used these high-throughput techniques to identify new subclasses of biomarkers (7,8), classify subtypes (9) and predict the outcome of human cancer (10–13). Gene expression profiling from microarray studies has been used to understand the development mechanism of human diseases. However, the majority of studies with regard to the identification of biomarkers have focused on mRNA and proteins. Compared with mRNA and proteins, microRNAs (miRNAs) are more likely to act as disease biomarkers due to their stable structure and easy detection (13). The abnormal expression of miRNAs is key in the progression of human cancer and may act as a biomarker that is used for a clinical diagnosis of early GC.

The present study identified two signature miRNAs, hsa-miR-196a and hsa-miR-148a, using the microarray technique, bioinformatics methods and biological experiment methods based on a group of clinical samples from Chinese patients. This single signature may potentially act as candidate biomarker for the early diagnosis of GC.

## Materials and methods

### Clinical samples

The clinical samples were collected from the Wuhan General Hospital of Guangzhou Command (Guangzhou, China). Information regarding the clinicopathological, therapeutic and outcome parameters of patients that were treated between August 2010 and December 2011 was collected retrospectively. Cancer staging was performed according to the fifth edition of the American Joint Commission on Cancer TNM criteria in 2000. All cancer samples were obtained from surgical specimens and all patients provided written consent for the use of these tissues for research purposes. A total of 86 patients were selected for the present study, including 44 samples from early GC patients and 42 normal gastric mucosa samples from non-cancer patients, which were used as a control group. The details of the patients that were used in this study are shown in Table I. The study was approved by the ethics committee of Wuhan General Hospital of Guangzhou Command (Wuchang, China). Written informed consent was obtained from the patients.

### miRNA microarray

The miRNA microarray analysis was performed as described in detail on the website of the Shanghai Biotechnology Corporation (http://www.ebioservice.com/). Briefly, 50–100 μg total RNA was used to extract the miRNAs using an miRNA isolation kit (AM1560; Ambion, Carlsbad, CA, USA). Fluorescein-labeled miRNAs were used for hybridization on an Affymetrix miRNA chip 2.0 (Affymetrix, Santa Clara, CA, USA). The fluorescence signals were scanned using a GeneChip Scanner 3000 7G (Affymetrix). The raw data were normalized and analyzed using GeneChip Command Console 1.1 software (Affymetrix).

### RNA extraction and quantitative PCR (qPCR)

RNA was extracted from larynx carcinoma and normal esophageal mucosa tissues using TRIzol reagent (Invitrogen, Carlsbad, CA, USA), according to standard procedure. Mature miRNA sequences were acquired from the Sanger Institute miRBase Sequence Database (http://microrna.sanger.ac.uk/sequences/). Stem-loop reverse transcription primers for miRNAs were designed according to Chen *et al* (14). The reverse transcription reaction conditions that were used involved incubation at 16°C for 30 min, 42°C for 30 min and 72°C for 10 min. The thermal cycling procedure for the PCR involved an initial denaturation step at 95°C for 4 min, followed by 40 cycles at 95°C for 30 sec, 57°C for 30 sec and 72°C for 30 sec. The melt curves for each PCR were carefully analyzed to determine any non-specific amplification. The expression of each miRNA was calculated using the 2^−ΔΔCT^ formula and normalized to U6 snRNA expression (15).

### Bioinformatics algorithms

The significant analysis of microarray (SAM) method was used to perform the unsupervised calculation. The statistical technique is based on a t-test for finding significant genes in a set of microarray experiments and was proposed by Tusher *et al* (16). A hierarchical clustering of the differentially expressed genes was performed with Cluster 3.0 (http://bonsai.hgc.jp/~mdehoon/software/cluster/software) version using the average linkage algorithm. The top scoring pair (TSP) algorithm was used to perform the supervised calculation (17). The basic principle of the k-TSP is to identify miRNA pairs that are oppositely expressed (one upregulated and one downregulated) in two classes. All numerical analyses that are presented were performed using Matlab 7.0 (MathWorks Company, Natick, MA, USA).

### Receiver operating characteristic (ROC) curves and statistical analysis

The ROC curve analysis was conducted using the MedCalc software packages (version 8.2.1.0; MedCalc, Mariakerke, Belgium). The area under the curve (AUC) provided a measure of the overall performance of the diagnostic test. The ratio of the miRNA signal intensities and Ct value of each miRNA were used for the ROC calculation of the samples. The clinical data were analyzed using the t-test. The cumulative survival curve was compared using the log-rank test. P<0.05 was considered to indicate a statistically significant difference.

### miRNA-targeted gene prediction and signal pathway analyses

An miRNA target gene prediction database TargetScan 5.2 (http://www.targetscan.org) was used to predict the plausible targets of the miRNAs. An integrated gene ontology database molecular annotation system (MAS 3.0; http://www.capitalbio.com) was used to investigate the miRNA-targeted genes and their involvement in various signal pathways.

## Results

### Differentially expressed miRNA profiling

**Figure 1:**
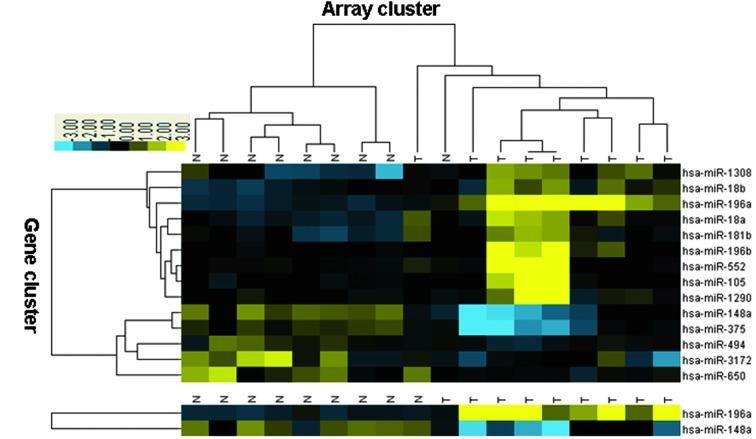
Cluster analysis of expressed miRNAs in early GC and normal samples. A total of 14 differentially expressed miRNAs, including nine upregulated and five downregulated miRNAs, were of significance in the early GC samples (according to the criteria of fold change >2; q=0). The columns represent samples and the rows represent miRNAs (black, yellow and blue correspond to unchanged, downregulated and upregulated, respectively). miRNA, micro RNA; GC, gastric cancer.

SAM was used to compare the expression data of nine early GC samples with nine normal samples. A total of nine upregulated and five downregulated miRNAs were identified with statistical significance in the early GC samples (Fig. 1A). The 14-miRNA profile may be used to differentiate between the cancer and normal samples with a classification accuracy of 94.4%. Furthermore, the TSP algorithm was used to identify the most efficient marker based on the 14-miRNA profile data. hsa-miR-196a and hsa-miR-148a were calculated to be the most efficient markers for classifying early GC and normal samples (Fig. 1B).

### qPCR validation

**Figure 2:**
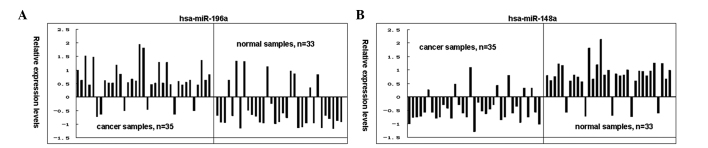
Quantitative PCR validation. (A) hsa-miR-196a was upregulated in 29 of the 35 GC samples and downregulated in 25 of 33 normal samples. (B) hsa-miR-148a was upregulated in 28 of 33 normal samples and downregulated in 28 of 35 GC samples. miR, microRNA; GC, gastric cancer.

**Figure 3:**
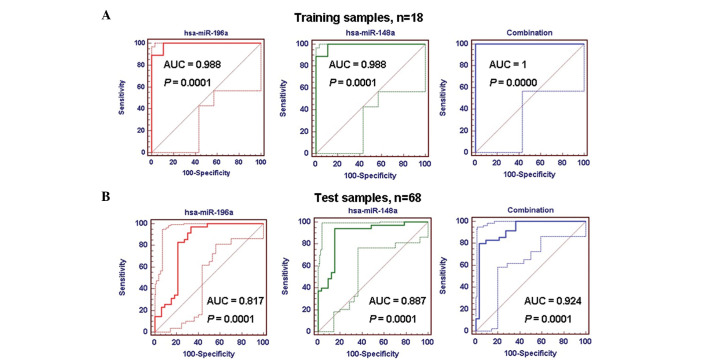
ROC analyses of the candidate biomarkers. (A) AUC value of the marker (combined hsa-miR-196a and hsa-miR-148a) was 1.0 in the training samples, which was higher than that of hsa-miR-196a or hsa-miR-148a alone. (B) AUC value of the marker was 0.924 in the test samples, which was also higher than that of hsa-miR-196a or hsa-miR-148a alone. This marker was more sensitive and specific for differentiating between the GC and normal samples. ROC, receiver operating characteristic; AUC, area under the curve, miR, microRNA; GC, gastric cancer.

The relative expression levels of hsa-miR-196a and hsa-miR-148a were detected in 68 test samples. The relative expression levels of this group of selected miRNAs obtained from the microarray data were consistently confirmed using qPCR analyses. hsa-miR-196a was upregulated in 29 of the 35 GC samples, with a total positive rate of 82.86%; whereas hsa-miR-196a was downregulated in 25 of the 33 normal samples, with a positive rate of 75.76% (Fig. 2A). hsa-miR-148a was upregulated in 28 of the 33 normal samples, with a positive rate of 84.85%; while it was downregulated in 28 of the 35 GC samples, with a positive rate of 80.00% (Fig. 3B).

### ROC curve analyses

ROC curves were used to analyze the classification sensitivity and specificity of the candidate biomarkers. hsa-miR-196a and hsa-miR-148a were combined to form one marker for this study. The present data revealed that the AUC value of the marker (combined hsa-miR-196a and hsa-miR-148a) was 1.0 in training samples, which was higher than that of hsa-miR-196a (0.988) or hsa-miR-148a (0.988) alone (Table II; Fig. 3A–C). Similar results were observed in the test samples; the AUC value of the marker in the test samples was 0.924, which was higher than that of hsa-miR-196a (0.817) or hsa-miR-148a (0.887) alone, and was more sensitive (80%) and specific (96.97%) for the classification of GC and normal samples (Table II; Fig. 3D–F).

### Signaling pathway analyses

In order to investigate the possible regulatory mechanisms of hsa-miR-196a and hsa-miR-148a in the process of early GC, the plausible targets were predicted using a bioinformatics database (TargetScan 5.2). A total of 211 genes were predicted to be the target genes of hsa-miR-196a. Signaling pathway analyses revealed that the majority of the targeted genes that were regulated by hsa-miR-196a were involved in pathways including ErbB, mTOR, MAPK, cell cycle, Jak-STAT, p53 and VEGF signaling pathways (Table III). A total of 536 genes were predicted to be the target genes of hsa-miR-148a. The targeted genes that were regulated by hsa-miR-148a were involved in the same pathways as hsa-miR-196a, with the exception of Wnt and TGF-β signaling pathways, which were regulated by hsa-miR-148a, but not by hsa-miR-196a (Table III).

## Discussion

High-throughput microarray experiments were the first step in the present study. The method has developed significantly and has become a comprehensive technique to aid in improving the understanding of cancer (18). The detection of all the known and unknown miRNAs in the human genome was easy in the present study through the use of microarray. The primary cancer cases were analyzed in order to identify the candidate biomarkers for early GC based on the microarray data. Finally, two miRNAs (hsa-miR-196a and hsa-miR-148a) were grouped as a signature with high sensitivity and specificity for differentiating between GC and normal samples, and may be a potential marker for the early diagnosis of GC.

miRNAs range in size from 19–25 nt and are protected by the RNA-induced silencing complex, which may render them less susceptible to RNA degradation compared with mRNA in these tissues. In addition, miRNA expression is able to be detected in blood samples, which is an excellent source for clinical studies. In the present study, a concise machine learning algorithm, TSP, was used for data-mining and selecting feature miRNAs based on the early GC microarray data. This TSP method has been well-used by other studies in biomarker identification for human diseases (19). Finally, the candidate biomarkers were validated in the laboratory by qPCR.

Studies have shown that miR-196a is upregulated in human cancer, including GC, and promotes the cell proliferation process (20–22). miR-196a may act as a candidate biomarker for GC (23). Other studies have shown miR-196a to contribute to the risk of carcinoma, metastasis and recurrence and to be associated with risk and prognosis by the regulation of its target genes (24–26). The present results are consistent with the majority of studies that describe miR-196a to be highly expressed in GC. A low expression of miR-148a has also been confirmed in certain human cancers and was associated with the cancer patient’s prognosis by regulating its target genes (27–29). miR-148a may act as candidate biomarker in human cancer (30,31). However, no studies are available with regard to the combination of the two miRNAs as a signature for diagnosis or prognosis in human cancer. Although the key involvement of miR-196a and miR-148a in GC are unclear, the present data are encouraging.

The current study revealed that certain cancer-related pathways, including ErbB, mTOR, MAPK, cell cycle, Jak-STAT, p53 and VEGF signaling pathways, were regulated by both miR-196a and miR-148a. However, the present data also revealed that two significant pathways involved in carcinogenesis, Wnt and TGF-β, were regulated by miR-148a, but not by miR-196a. These multiple signal pathway alterations, particularly those that include the Wnt and TGF-β pathways, may reasonably affect the progress of GC carcinogenesis. The SMAD2 gene is significant in the two pathways and was regulated by miR-148a, as shown by the bioinformatics analyses. Therefore, we propose that miR-148a may be a key regulator in the development of early GC by regulating the SMAD2 gene and participating in the Wnt and TGF-β pathways. However, further confirmation of this in the laboratory is required.

In summary, two miRNAs were identified that were differentially expressed in early GC compared with normal samples. By combining the two miRNAs as a single signature, differentiating between cancer and normal samples may be more accurate. The two miRNAs may act as candidate biomarkers for early GC.

## Figures and Tables

**Table I:** Details of the patients that were used in this study.

Characteristic	Cancer group (n=44)	Control group (n=42)	P-value
Gender, n			0.976
Male	25	24	
Female	19	18	
Age, years			0.343
Median	55	51	
Range	37–78	32–74	
Stage, n			-
I	13	-	
II	31	-	
Patient status, n			0.105
Survival	39	41	
Mortality	5	1	

**Table II:** ROC curve analyses of the biomarkers in the training and test samples.

Samples	Classifiers	Sensitivity (%)	Specificity (%)	AUC	95% CI	P-value
Training (n=18)	hsa-miR-196a	100.00	88.89	0.988	0.792–1.000	0.0001
	hsa-miR-148a	88.89	100.00	0.988	0.792–1.000	0.0001
	Combination	100.00	100.00	1.000	0.575–0.947	0.0000
Test (n=68)	hsa-miR-196a	97.14	66.67	0.817	0.705–0.901	0.0001
	hsa-miR-148a	94.29	84.85	0.887	0.787–0.951	0.0001
	Combination	80.00	96.97	0.924	0.833–0.974	0.0001

ROC, receiver operating characteristic; AUC, area under the curve; CI, confidence interval; miR, microRNA.

**Table III:** Signaling pathway analyses of genes regulated by hsa-miR-196a and hsa-miR-148a.

	hsa-miR-196a		hsa-miR-148a	
		
Pathway	Gene	q-value	Gene	q-value
ErbB signaling	NRAS, ABL1, CDKN1B, ABL2	3.26×10^−4^	SOS2, TGFA, SOS1, ERBB3, NRAS, ABL2, PIK3R3, CAMK2A	2.70×10^−6^
mTOR signaling	RICTOR, TSC1, IGF1	8.32×10^−4^	IGF1, RICTOR, PRKAA1, PDK1, PIK3R3	1.26×10^−4^
MAPK signaling	NRAS, MAP3K1, RASGRP1, MAP4K3, PDGFRA	1.63×10^−3^	SOS2, GADD45A, SOS1, MAP3K4, MRAS, NRAS, CDC25B, NLK	1.97×10^−3^
Cell cycle	ABL1, CDKN1B, CDC25A	4.52×10^−3^	YWHAB, CDC14A, GADD45A, SKP1, CDK6, SMAD2, CDC25B, E2F3	1.90×10^−5^
Jak-STAT signaling	OSMR, SOCS4	2.79×10^−2^	SOS2, SOS1, PIK3R3, SOCS3	2.43×10^−2^
p53 signaling	IGF1	5.43×10^−2^	PTEN, IGF1, GADD45A, SERPINE1, CDK6	4.16×10^−4^
VEGF signaling	NRAS	5.86×10^−2^	NFAT5, NRAS;PIK3R3	1.70×10^−2^
Wnt signaling			NFAT5, WNT1, ROCK1, PRICKLE2, TBL1XR1, SKP1, WNT10B, VANGL1, CAMK2A, PSEN1, SMAD2, NLK, PPARD,	5.00×10^−9^
TGF-β signaling			INHBB, ROCK1, NOG, ACVR1, SKP1, GDF6, LTBP1, ACVR2B, SMAD2, SP1	2.36×10^−8^

miR, microRNA.
